# Blockchain and medicine: From digital promise to frontline practice

**DOI:** 10.1016/j.amsu.2022.103555

**Published:** 2022-04-02

**Authors:** Armin Abadeh

**Affiliations:** University of Toronto, Department of Medicine, Canada

## Abstract

The promise of blockchain has far-reaching ramifications for health-care stakeholders. Blockchain technology has the potential to improve health care by putting the patient at the centre of the system and improving health data security, privacy, and interoperability. This is a reflective piece to highlight the opportunities for applications of blockchain technology in the future of healthcare.

Against the backdrop of an ongoing pandemic, global inequities have become magnified, healthcare systems are perpetually strained, and, for many clinicians, preventative care has gone from being the standard to an aspiration. Beyond exposing critical capacity issues, the COVID-19 pandemic has magnified injustices that cut across socioeconomic lines and has increased vulnerabilities. It has challenged our norms around therapeutics and has given rise to a host of ethical dilemmas, from accelerating clinical trials to global vaccine distribution. One unexpected silver lining is that the increased uptake of telemedicine outside of rural communities has encouraged broader discussions on innovations. Personalized medicine, gene editing, 3D printed organs, customized stem cell injections, and artificial intelligence are expected to play a significant role in improving community outcomes and advancing the medical field in the coming decades. Although distributed ledgers and blockchain technology may appear to be irrelevant to the current daily tasks of healthcare professionals, there's a reason why “NFT” (non-fungible token) not only made it into Collins Dictionary in 2021 but became the word of the year. Comparable to virtual art collections, NFTs are digital assets that can be created, encrypted, and transferred from one person to another using blockchain technology. In what follows, I will outline the parallels between NFTs and medicine that can serve as both an inspiration and a call to action to integrate blockchain technology into healthcare to streamline administrative processes, reduce costs, increase accessibility, and prevent data breaches.

Essentially, blockchain-enabled smart contracts are immutable, transparent, decentralized, borderless, and require no intermediaries for authorization. While blockchain technology has become a critical priority for the largest organizations in the world and is recognized by Bloomberg as a nearly $41B industry, to my knowledge this topic has not made it into a lecture in medical school or grand rounds. As a physician working in the midst of a global pandemic, I cannot recall hearing anything about how blockchain is being used to tackle COVID-19 through early detection or fast-tracking drug trials. With respect to non-fungible tokens, their ownership is a function of their authenticity. Like the genetic make-up of each patient, NFTs are comprised of unique combinations of characters, with no duplicates – meaning they are digital certificates that an asset is one-of-a-kind. Just as NFT collectors value the originality of their digital assets, the best medical providers focus on the entire patient picture. Socioeconomic status, race, religious preferences, ethnic norms, health literacy, and lifestyle are critical to understanding the unique needs of each person in our exam room (or on our computer screen in the age of COVID-19).

On that note, it is important to underscore that NFTs cannot be divided into smaller pieces. When it comes to images, every pixel is accounted for in the unique blockchain coding. Similarly in medicine, context is what confers meaning. This is perhaps even more apparent in counseling, psychiatry, and social work settings, where there is a strong evidence base on the connection between social inequities, low socioeconomic status, and poor mental health outcomes. Consider a single mother, who is getting by on welfare, lives in an underserved neighborhood, and is caring for two teenagers with diabetes. Her medical history – which is shaped by her daily experiences – would be incomplete if you were to simply ask her to describe her symptoms. Under the traditional medical model, what is *noteworthy* from a clinical standpoint is only part of the equation. As physicians, we must look beyond mere symptomology with each new patient we encounter to identify the biopsychosocial dimensions of their healthcare and well-being, and provide then with the holistic care they need to not only survive, but to thrive. By extension, the visible aspects of an NFT can be easily misunderstood without researching their intrinsic and extrinsic value.

For the past two decades, “whole person” or “holistic” care has been ingrained in every physician's mind. And I would argue that the uninitiated, ‘untrained’ NFT collectors are viewing their assets similarly. The same lens that reveals the uniqueness, diversity, and indivisibility of NFTs is what we use in our day-to-day patient interactions. Yet, this philosophical approach – of respecting the integrity of the whole – has only entered into mainstream discussions and popular culture recently, with the understanding that NFTs are built upon and derive their value and meaning from blockchain technology. Looking ahead, blockchain technology can be leveraged to create a standard for communications and collaborations between our healthcare system's key stakeholders – allowing for interoperability on a scale that has never been seen before. Blockchain technology promises to heal our fragmented system by facilitating health information transfer, enhanced data transparency, improved patient care, efficiency, and synergistic research. With this infrastructural transformation, there will be significant challenges to overcome in the long road ahead, but its potential is unparalleled and well worth the effort.

